# MSA-DET: A Multi-Scale Attention Network with Adaptive Feature Fusion for SAR Ship Detection

**DOI:** 10.3390/s26133970

**Published:** 2026-06-23

**Authors:** Sai Wan, Zhiyong Tao, Lu Chen

**Affiliations:** 1 School of Electronic and Information Engineering, Liaoning Technical University, Huludao 125105, China; 2 Erdos Research Institute, Liaoning Technical University, Erdos 017004, China

**Keywords:** SAR ship detection, YOLOv11, attention mechanism, feature fusion, multi-scale attention, sparse self-attention, feature pyramid, object detection, remote sensing, deep learning

## Abstract

Synthetic aperture radar (SAR) ship detection faces three persistent challenges: coherent speckle noise that obscures target boundaries, heterogeneous background clutter in coastal and harbor scenes, and ship targets whose spatial extent varies by more than an order of magnitude within the same image. To address these issues jointly, this paper proposes MSA-DET, an improved SAR ship detection network built upon YOLOv11. In the backbone, a Multi-Scale Cross-axis Attention module (MSCAttention) runs horizontal and vertical axial attention branches in parallel across multiple receptive-field scales, sharpening feature representations for ship targets that vary widely in size and orientation. In the neck, the standard C3k2 block is redesigned as C3k2_SSA by embedding sparse self-attention, which selectively focuses on the most discriminative spatial tokens while suppressing speckle interference and reducing computational overhead. An Adaptive Spatial Feature Fusion detection head (ASFF) replaces fixed pyramid-level aggregation with learned per-pixel blending weights, resolving gradient conflicts across scales and improving localization consistency for both small and large ships. On the HRSID dataset, MSA-DET achieves an mAP@0.5:0.95 of 63.6% and mAP@0.5 of 88.1%, representing gains of 4.0% and 1.6% over the YOLOv11n baseline; on SSDD, it reaches 69.6% and 97.7%, surpassing the baseline by 7.2% and 2.1%, respectively. These results demonstrate that coordinated multi-stage redesign—rather than isolated module substitution—is an effective strategy for SAR-oriented ship detection. The accuracy gains are accompanied by a moderate increase in model size (8.9 M parameters versus 2.6 M for YOLOv11n) and computational cost (9.6 G FLOPs versus 6.3 G), a trade-off that is justified by the substantial improvement in detection quality.

## 1. Introduction

Synthetic aperture radar (SAR) enables round-the-clock, all-weather imaging of the ocean surface, making it a cornerstone technology for maritime surveillance, vessel traffic monitoring, and illegal fishing detection [1,2,3,4]. Despite these advantages, SAR ship detection is substantially harder than its optical counterpart. Three factors are primarily responsible. First, coherent speckle noise—a multiplicative interference pattern inherent to SAR imaging—degrades contrast between ship echoes and the sea background [5,6]. Second, the spatial footprint of ships varies enormously: in the HRSID benchmark, small vessels account for 54.5% of all annotated instances [7], while a typical ship occupies less than 4% of the image area [8]. Third, densely berthed harbor scenes create severe inter-target overlap and clutter that makes individual ship segmentation unreliable. Together, these factors demand a detector that is simultaneously noise-robust, scale-adaptive, and capable of resolving closely spaced targets.

Classical constant false-alarm rate (CFAR) detectors [9,10,11] address the noise problem through statistical sea-clutter modeling, but their rigid background assumptions break down in heavy weather, nearshore environments, and any scenario that deviates from their assumed clutter distribution [12,13]. Deep convolutional networks largely overcame these limitations: Li et al. [14] applied Faster R-CNN to SAR ship detection and demonstrated that learned feature representations could substantially outperform hand-crafted CFAR statistics. The YOLO family subsequently became the dominant paradigm for this task, trading some accuracy for dramatic inference speedups that approach real-time performance [15,16,17]. The latest release, YOLOv11, further strengthens the nano-scale architecture with C3k2 bottleneck modules, spatial pyramid pooling (SPPF), and C2PSA attention blocks [18], establishing a strong modern baseline for SAR-oriented research.

Despite this progress, two structural gaps remain. First, existing attention modules—SE-Net, CBAM, and ECA-Net [19,20,21]—operate at a single scale and were designed for optical images with low speckle noise; applied to SAR imagery, they tend to amplify background responses rather than isolate ship echoes [22,23,24]. Second, standard feature pyramid fusion strategies (FPN and PANet [25,26]) merge multi-scale features with fixed, spatially uniform weights. In harbor scenes where small fishing boats and large cargo vessels occupy the same image, fixed fusion weights cause gradient conflicts during training and limit detection consistency across scales [27,28]. Correcting both problems simultaneously—scale-aware noise suppression together with adaptive multi-scale fusion—is the central challenge that motivates this work.

To address these gaps, we propose MSA-DET, a YOLOv11-based detector whose backbone, neck, and detection head are each redesigned with a specific SAR-oriented goal. The three modifications—MSCAttention, C3k2_SSA, and ASFF—are independently motivated by different failure modes of the baseline and are designed to cooperate as an integrated system rather than as isolated plug-ins. The novelty of this work lies not in any single component in isolation, but in (i) the principled adaptation of each technique to the SAR noise and scale regime, and (ii) the demonstration that coordinating all three stages of the pipeline produces accuracy gains that no single-stage improvement can match. The main contributions are as follows:

1. A unified multi-scale detection framework, MSA-DET, that simultaneously addresses scale imbalance and speckle noise suppression for SAR ship detection through coordinated modifications to the backbone, neck, and detection head.
2. The domain adaptation of Multi-Scale Cross-axis Attention (MSCAttention) [29] from medical image segmentation to the SAR detection backbone: its parallel multi-scale axial branches and cross-dimensional coupling are shown to be effective for discriminating ship targets that vary widely in orientation and size under SAR speckle conditions.
3. The integration of Sparse Self-Attention (SSA) [30] into a C3k2 CSP bottleneck (C3k2_SSA), coupling convolutional feature extraction with global sparse attention in a single neck block, with selective deployment at the P3 and P4 feature levels to broaden effective context while suppressing speckle-dominated background tokens at sub-quadratic cost.
4. The replacement of the YOLOv11 detection head with an ASFF-based head [27] that pairs anchor-free DFL regression and depthwise-convolutional classification branches with learned, per-pixel Softmax fusion weights, resolving scale-induced gradient conflicts that are particularly pronounced in SAR harbor scenes.
5. Thorough ablation and comparative experiments on the HRSID and SSDD benchmarks, demonstrating mAP@0.5:0.95 improvements of 4.0% and 7.2% over YOLOv11n with a moderate computational overhead.

The rest of this paper is organized as follows. Section 2 surveys related work. Section 3 details the MSA-DET architecture and the SAR-motivated design rationale of each module. Section 4 presents the experimental setup, ablation study, comparative results, and visualizations. Section 5 draws conclusions and outlines future directions.

## 2. Related Work

The YOLO family has evolved into the dominant single-stage detector for SAR ship detection, owing to its compact architecture and near-real-time inference speed. YOLOv11, the most recent edition, introduces C3k2 bottleneck modules, a C2PSA context-aggregation block, and a refined task-aligned loss that collectively improve accuracy without compromising throughput [18].

**Feature extraction and hybrid CNN methods.** Early deep learning work on SAR targets combined classical hand-crafted features with CNNs to improve robustness against speckle. Ai et al. [31] integrated multi-scale rotation-invariant Haar-like texture descriptors into a CNN pipeline, exploiting the directional statistics of SAR clutter for ship detection in multi-target scenes. Ai et al. [32] proposed a multi-kernel-size feature fusion CNN that processes SAR target patches in parallel with kernels of sizes 3×3, 5×5, and 7×7, capturing both local texture and global contour cues before optimal fusion. Although these hybrid methods outperformed pure CFAR detectors by a substantial margin, their reliance on fixed feature priors and patch-based processing limits scalability and generalization across sensor configurations.

**Multi-scale feature enhancement.** Zhu and Miao [33] proposed YOLOv7-LDS, pairing squeeze-and-excitation attention with a depthwise-separable ELAN module to enrich multi-scale representations at reduced computational cost; however, the cross-level fusion strategy remains static. Wei et al. [34] appended an Efficient Multi-Scale Attention (EMA) block at the tail of the SED-YOLO backbone, improving small-target recall at the cost of limited inter-scale interaction.

**Attention and context modeling.** Zhao et al. [35] adapted DETR with group-axial attention for directional target cues, though the transformer backbone carries a heavy computational footprint. Chen et al. [36] widened the receptive field of coordinate attention through expand-fold operations (PEA module), yet provided no dedicated mechanism against SAR speckle. For multi-modal SAR scenarios, Xue et al. [37] proposed LMCNet, a lightweight modality compensation network that uses knowledge distillation to train a SAR-only student model from a multimodal (SAR + AIS) teacher, demonstrating that explicit inter-modality compensation substantially reduces false alarms in cluttered coastal areas even when auxiliary data is unavailable at inference time.

**Feature fusion and detection head design.** Doherty et al. [38] integrated a bidirectional feature pyramid (BiFPN) into a YOLO backbone; the learnable aggregation weights improve multi-scale accuracy but remain spatially uniform—every pixel at a given scale receives the same blending ratio. Zhang et al. [39] introduced AALFF for adaptive adjacent-layer fusion, though the design targets blurred optical boundaries rather than the high-contrast, low-area signatures of SAR ships. Wang et al. [40] combined multi-scale sequence fusion with channel attention in DSP-YOLO, yet the detection head still applies fixed, scale-invariant fusion weights.

Taken together, these methods address backbone, neck, or detection heads in isolation. Table 1 summarizes the component coverage and key limitations of representative prior methods. No prior work ties all three stages into a coherent noise-suppressing, scale-adaptive pipeline. MSA-DET fills this gap by engineering each stage for a distinct SAR failure mode and demonstrating that coordinating them yields compounding accuracy gains that no single-stage modification achieves alone.

## 3. Materials and Methods

**Figure 1:**
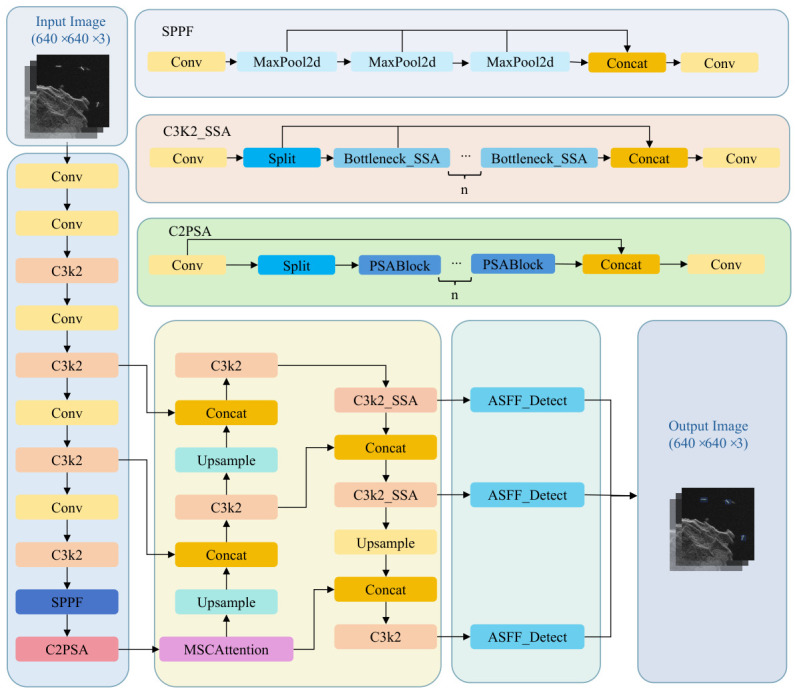
Overallarchitecture of MSA-DET. MSCAttention is inserted at Layer 11 (backbone, after SPPF and C2PSA); C3k2_SSA replaces standard C3k2 at neck Layers 14, 17, and 20 (P4/P3/P4); Layer 23 retains standard C3k2 for P5; three ASFF-Head instances fuse features at P3/P4/P5 with learned per-pixel weights. The ellipsis (“…”) denotes standard YOLOv11 backbone blocks that are omitted for visual clarity; all modified modules and their layer indices are shown explicitly. Details of each module are described in Section 3.

Figure 1 gives an overview of the MSA-DET architecture. The detector builds on YOLOv11 and introduces three coordinated modifications, each targeting a different bottleneck in SAR ship detection. All three modules draw on existing techniques that are adapted to the SAR context in specific ways: MSCAttention is originally proposed for medical image segmentation [29] and is here repurposed for SAR by placing it at the deepest backbone stage (after Stage 4) where receptive-field diversity is most needed for multi-scale ship echoes; C3k2_SSA adapts the SparseViT attention [30] from image manipulation localization into a CSP bottleneck, exploiting its sparsification property specifically to zero-weight speckle-dominated background tokens; and ASFF [27] is applied here to replace the YOLOv11 detection head with learnable per-pixel fusion weights, addressing the scale inconsistency problem that is particularly pronounced in SAR harbor scenes. The modifications are described below, with explicit comparison to the original published implementations where relevant.

1. **Backbone Enhancement.** A Multi-Scale Cross-axis Attention (MSCAttention) module is inserted after the SPPF and C2PSA blocks. By exchanging information across two orthogonal axial branches, this module sharpens feature representations for ship targets that vary widely in scale and orientation.
2. **Neck Optimization.** The neck replaces standard blocks with the C3k2_SSA module, which pairs convolution with sparse self-attention in parallel. This combination broadens the effective context window and suppresses complex background noise and sea clutter without a prohibitive increase in computation.
3. **Head Adaptation.** An Adaptive Spatial Feature Fusion (ASFF) detection head replaces the original output stage. ASFF learns position-dependent fusion weights for multi-scale features, tightening localization for ships of diverse sizes.

These three modifications work as an integrated whole rather than independent add-ons. The sections that follow describe the design rationale behind each module in detail; comparative and ablation experiments then quantify both the individual and combined effects of these components.

### 3.1. MSCAttention Module

Standard self-attention captures global context but scales quadratically, $O({\left(HW\right)}^{2})$, a cost that becomes prohibitive on high-resolution SAR feature maps [42,43]. Axial Attention brings this down to O(HW(H+W)) by decomposing 2D attention into successive horizontal and vertical passes [43]. The sequential nature of that decomposition, though, limits how much the two spatial dimensions can interact with each other. Most existing attention designs also process features at a single scale [42,44]—a poor match for SAR images where ship targets range from small fishing boats to large cargo vessels within the same scene.

We address both shortcomings by adapting the cross-axis attention mechanism from MCANet [29] into the SAR detection backbone. MCANet’s decode head attention runs two axial branches in parallel—each aggregating multi-scale 1D strip features along one axis—and couples them cross-dimensionally by routing each branch’s queries from the opposite axis’s feature map. The kernel configuration (1 × 7, 1 × 11, 1 × 21 horizontal and their vertical counterparts), the depthwise-separable grouping, and the cross-axis Q-K-V coupling are inherited directly from that design. Our contribution is the domain transfer: transplanting this mechanism from a medical image segmentation decoder, where it was designed for pixel-level boundary discrimination, to the deepest stage of a SAR object detection backbone, where its multi-scale receptive fields and bidirectional spatial coupling address the scale and orientation diversity of ship echoes in cluttered SAR scenes. MSCAttention is inserted at Layer 11, after the backbone has applied five stride-2 downsampling operations that reduce the 640×640 input to a 20×20 feature map (1/32 of the input resolution); operating at this compressed spatial scale keeps the attention overhead modest while allowing the module to capture global ship-level context.

**Figure 2:**
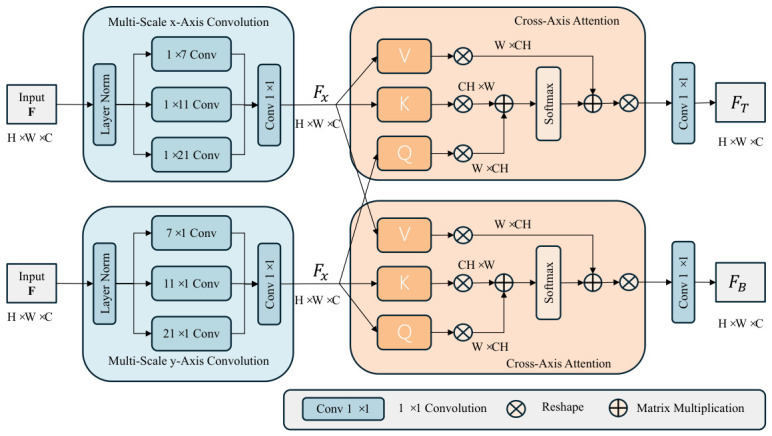
Internalmechanism of MSCAttention. Multi-scale 1D convolutions extract spatial features along two orthogonal axes; cross-axis attention then enables bidirectional information exchange between horizontal and vertical dimensions.

The internal structure is shown in Figure 2. Given an input feature map $F\in R^{H\times W\times C}$ (where *H* and *W* are the spatial height and width of the feature map in pixels, and *C* is the number of channels), the module splits processing into an *x*-axis branch and a *y*-axis branch. Each branch applies three 1D convolution kernels of different sizes (*x*-axis: 1×7, 1×11, 1×21; *y*-axis: 7×1, 11×1, 21×1) to the layer-normalized input. The three kernel sizes are chosen to cover three distinct receptive-field regimes on the 20×20 feature map (1/32 of the input resolution): 7-pixel kernels target short-range context corresponding to small vessels, 11-pixel kernels capture mid-range context for medium-sized ships, and 21-pixel kernels provide long-range context for large targets; together they span the full scale range of SAR ship echoes observed in HRSID and SSDD without requiring separate branches per target category. In the *x*-axis branch the multi-scale outputs are summed and compressed through a 1×1 convolution:

(1) $$F_{x}={\mathrm{Conv}}_{1\times 1}\left(\sum_{i=0}^{2}{\mathrm{Conv}1D}_{x}^{i}\left(\mathrm{Norm}\left(F\right)\right)\right)$$

The *y*-axis branch produces $F_{y}$ in a mirror fashion. Cross-axis attention is then constructed as follows. For the horizontal branch, Keys (*K*) and Values (*V*) come from $F_{x}$ while Queries (*Q*) come from $F_{y}$; dot-product similarity is computed, normalized with Softmax, and used to weight *V*. A residual connection yields the horizontal cross-axis feature $F_{T}$. The vertical branch reverses the roles: *Q* is drawn from $F_{x}$, and K,V from $F_{y}$, producing $F_{B}$. Eight attention heads operate in parallel to widen the representational capacity. Because each branch’s queries originate in the opposite axis, the module captures global dependencies along both directions simultaneously—something sequential axial attention cannot achieve. The final output folds the two refined branches back together with the original input through a residual path:

(2) $$F_{\mathrm{out}}={\mathrm{Conv}}_{1\times 1}\left(F_{T}\right)+{\mathrm{Conv}}_{1\times 1}\left(F_{B}\right)+F$$

The three kernel widths—7, 11, and 21 pixels—are chosen to span the typical spatial extent of SAR ship targets: short-range (7-pixel) kernels capture fine structural details such as superstructure edges, medium-range (11-pixel) kernels cover the body width of small-to-medium vessels, and long-range (21-pixel) kernels address the full length of large cargo ships. These scales align with the bounding-box size distribution observed in HRSID, where ship lengths range roughly from 10 to 200 pixels at the native 800×800 resolution. The number of attention heads is set to h=8 following standard transformer practice [45]: with $d_{k}=C/8$ channels per head, each head captures a distinct subspace of the joint spatial representation, and 8 heads provide a good balance between representational diversity and computational efficiency.

Within MSA-DET, MSCAttention is placed at Layer 11—after the SPPF layer (Layer 9) and C2PSA block (Layer 10) at the deepest backbone stage. The YOLOv11 backbone applies five stride-2 downsampling steps (Layers 0, 1, 3, 5, and 7), so Layer 11 operates on 20×20 feature maps ($\frac{1}{32}$ of the 640×640 input). At this compact resolution the computational overhead of multi-scale cross-axis attention is modest, while the rich semantics of deep features and the large effective receptive fields are precisely what is needed to discriminate ship echoes from complex sea clutter.

### 3.2. C3k2_SSA Module

How well a detector extracts features largely determines its final accuracy. Conventional CNNs such as ResNet [46] are adept at encoding local texture, yet their fixed-geometry kernels cap the effective receptive field, leaving long-range spatial relationships under-represented. Vision Transformers (ViT) [47] remedy this through self-attention over the full feature map, but at $O(N^{2})$ cost—impractical once resolution climbs. Window-based variants like Swin Transformer [48] cut that cost by confining attention to local patches, though stitching information across windows adds its own design overhead.

**Figure 3:**
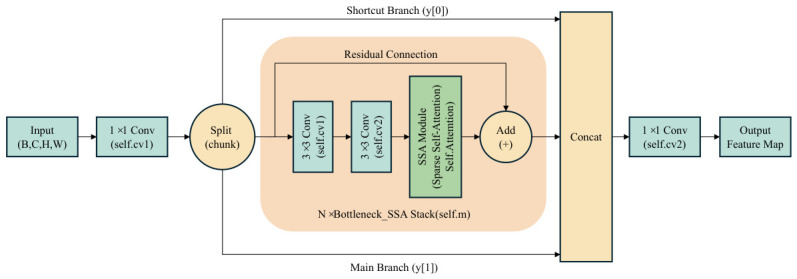
Architecture of C3k2_SSA. The CSPNet split-path structure is combined with Sparse Self-Attention to capture global dependencies efficiently while suppressing noise.

We seek a middle ground: global context at sub-quadratic expense. To that end, the Sparse Self-Attention (SSA) block of Su et al. [30] is integrated into the C3k2 CSP bottleneck, yielding the C3k2_SSA module depicted in Figure 3. The SSA block itself retains its original design from SparseViT [30]: layer-scale parameters initialized at $10^{-6}$ for training stability, a depthwise convolutional positional embedding (3×3, groups=dim), and fused linear projections for Q, K, and V. The contribution of C3k2_SSA is the integration strategy: SSA is appended to the two-convolution path of a C3k2 CSP bottleneck, creating a serial CNN-then-attention block that first extracts local texture with standard convolutions before applying sparse global attention. This hybrid is then selectively deployed at the P3 and P4 neck levels, while P5 retains a standard C3k2 block—a deliberate choice that concentrates sparse attention at the feature scales where small and medium ship discrimination is most critical.

The key idea is to let self-attention operate only on the most informative token pairs. For an input feature map $X\in R^{H\times W\times C}$ (notation consistent with Section 3.1; N=H×W denotes the total number of spatial tokens), linear projections first produce the familiar Query (*Q*), Key (*K*), and Value (*V*) matrices:

(3) $$Q=XW^{Q},\;K=XW^{K},\;V=XW^{V}$$

where $W^{Q},W^{K},W^{V}\in R^{C\times d_{k}}$ are learnable projection matrices and $d_{k}=C/h$ is the per-head dimension (*h* denotes the number of attention heads, set to 4 following the default configuration of the original SSA implementation [30]). Whereas standard attention computes dense pairwise scores, SSA applies a Top-*k* sparsification with k=⌊0.25N⌋ (retaining the top 25% of key positions per query, following [30]): for every query position *i*, only the *k* highest-scoring key positions *j* are retained; all other entries are masked to −∞, effectively zeroing their contribution after Softmax. The resulting sparse attention matrix *M* takes the form

(4) $$M_{ij}=\left\{\begin{array}{cc} \frac{q_{i}k_{j}^{⊤}}{\sqrt{d_{k}}} & \mathrm{if}\;\frac{q_{i}k_{j}^{⊤}}{\sqrt{d_{k}}}\in \text{Top-}k\left(A_{i,:}\right)\\ -\infty & \mathrm{otherwise} \end{array}\right.$$

Here $A_{i,:}$ collects all raw attention scores for the *i*-th query before sparsification. The attended output follows the usual weighted aggregation

(5) $$Y_{SSA}=\mathrm{Softmax}\left(M\right)V$$

A residual connection then merges $Y_{SSA}$ with the original input, preserving gradient flow and encouraging feature reuse. The net effect resembles a selective filter: the network attends to the few most relevant spatial locations—typically ship pixels and their immediate surroundings—while background clutter and speckle receive near-zero weight.

Inside MSA-DET, C3k2_SSA blocks are deployed at three neck positions: Layer 14 (P4, after concatenation with backbone P4 features), Layer 17 (P3, for small-target detection), and Layer 20 (P4, after bottom-up path aggregation). Layer 23 (P5, large-vessel scale) retains a standard C3k2 block, because large ships produce high-confidence detections even without sparse attention, and allocating SSA computation to the coarsest level yields diminishing returns. This selective deployment concentrates sparse attention where small and medium ship discrimination is most challenging, while keeping the overall FLOPs increase moderate.

### 3.3. ASFF-Head Module

Single-stage detectors built on feature pyramids usually merge information across scales with element-wise addition or simple concatenation [25,49]. Neither operation accounts for a fundamental mismatch: a ship that serves as a positive training sample at one pyramid level may overlap with pure background at another, creating gradient conflicts that confuse the optimizer [50]. Some work sidesteps this by masking out nearby-level regions [51], but such hand-crafted rules tend to produce false alarms at whichever level was suppressed. A more principled solution is to let the network decide, at every spatial position, how much each level should contribute. That is precisely what the Adaptive Spatial Feature Fusion (ASFF) detection head [27] does—it learns per-pixel fusion weights that filter out conflicting signals, with negligible extra latency.

**Figure 4:**
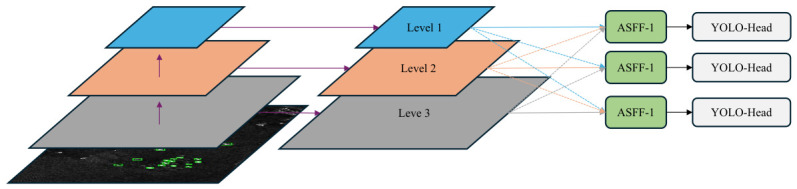
Architectureof the ASFF-Head. Per-pixel spatial weights learned via Softmax adaptively fuse features from different pyramid levels, resolving scale inconsistency. Arrow convention: solid arrows denote forward feature flow between modules; dashed arrows denote upsampling/downsampling operations used to align multi-scale features to a common resolution before adaptive fusion.

The mechanism operates as follows (see Figure 4). Three feature maps from the neck (Levels 1–3) are first aligned to a common spatial resolution: for a target level *l*, every other level’s features $x^{n}$ are resized through upsampling or downsampling to yield $x^{n\to l}$. Fusion then proceeds through learned spatial weights. Taking Level 1 as an illustration, the output at each position is

(6) $$y_{ij}^{l}=\alpha_{ij}^{l}\cdot x_{ij}^{1\to l}+\beta_{ij}^{l}\cdot x_{ij}^{2\to l}+\gamma_{ij}^{l}\cdot x_{ij}^{3\to l}$$

where $\alpha_{ij}^{l},\;\beta_{ij}^{l},\;\gamma_{ij}^{l}$ are non-negative weights that sum to one. They are parameterized via Softmax over learnable control variables:

(7) $$\alpha_{ij}^{l}=\frac{e^{\lambda_{\alpha ij}^{l}}}{e^{\lambda_{\alpha ij}^{l}}+e^{\lambda_{\beta ij}^{l}}+e^{\lambda_{\gamma ij}^{l}}}$$

The parameters $\lambda_{\alpha }^{l},\;\lambda_{\beta }^{l},\;\lambda_{\gamma }^{l}$ are produced by 1×1 convolution layers and updated through standard backpropagation. At positions where a genuine target exists, the weight for the most appropriate level gravitates toward one; at background positions, conflicting levels are driven toward zero. Gradient inconsistency across scales is therefore suppressed at its source [5].

Within MSA-DET, ASFF replaces the stock YOLOv11 detection head. The learned Softmax fusion weights are inherited from the original ASFF formulation [27]; the detection branches are updated to match YOLOv11’s anchor-free design: Distribution Focal Loss (DFL) regression and depthwise-separable convolutional classification heads replace the original YOLOv3-style anchor-based outputs. Three ASFF instances—ASFF-1, ASFF-2, and ASFF-3—sit at the P3, P4, and P5 output stages, each learning its own set of spatial fusion weights before passing the result to the classification and regression branches. The added FLOPs overhead is slight (3.3 G relative to the 6.3 G YOLOv11n baseline),yet the payoff is substantial: fused features carry less scale-induced noise, and targets of different sizes receive more consistent supervision. For SAR imagery, where small coastal vessels and large open-sea ships routinely coexist, this consistency matters a great deal.

## 4. Experiments

### 4.1. Datasets

We evaluate MSA-DET on two widely used SAR ship detection benchmarks—HRSID [7] and SSDD [8]—that together cover a broad spectrum of maritime conditions, from coastal harbors to open ocean, under different sea states and target scales. Table 2 summarizes the key specifications of each dataset.

**HRSID** [7] was the first benchmark designed explicitly for high-resolution SAR ship detection and instance segmentation. Its 5,604 images come from Sentinel-1B, TerraSAR-X, and TanDEM-X satellites at spatial resolutions between 0.5 and 3 m, and span all four standard polarization modes (HH, VV, HV, and VH). A total of 16,951 ship instances are annotated across these images, each of which is uniformly cropped to 800×800 pixels. We follow the established split of 65% for training and 35% for testing. Following the MS-COCO convention [52], we classify ship instances whose bounding-box area is smaller than $32^{2}=1024$ pixels^2^ (on the 640×640 input resolution) as *small targets*, and those larger than $96^{2}$ pixels^2^ as *large targets*. Under this definition, 54.5% of HRSID instances are small targets, underscoring the severe scale imbalance that the multi-scale design of MSA-DET is intended to address.

**Figure 5:**
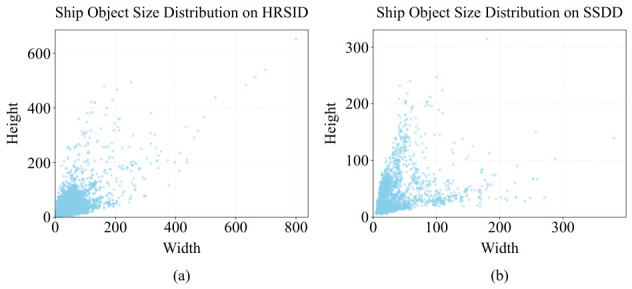
Jointdistribution of ship target scale (square root of bounding-box area normalized by image area) and aspect ratio for HRSID and SSDD. Each point represents one annotated ship instance; the two datasets are distinguished by color/marker style in the embedded legend. The wide spread along both axes underscores the multi-scale challenge that any SAR ship detector must handle.

**SSDD** [8], often called the “ImageNet” of SAR ship detection, gathers 1160 images from RadarSat-2, TerraSAR-X, and Sentinel-1 with resolutions spanning 1 to 15 m. The 2456 annotated ships appear under all four polarizations. Unlike HRSID, image dimensions in SSDD are not fixed: heights range from 190 to 526 pixels and widths from 214 to 668 pixels, introducing additional variation that tests a detector’s robustness. We adopt the official random 80/20 train–test partition for all experiments on this dataset; the split indices are fixed prior to any training run so that all compared methods operate on identical subsets. Figure 5 visualizes the joint distribution of ship target scales and aspect ratios across both datasets, confirming the severe scale imbalance that motivates the multi-scale design of MSA-DET.

### 4.2. Experimental Setup

All experiments ran on a workstation fitted with an Intel Xeon Platinum 8481C CPU, an NVIDIA RTX 4090 GPU (24 GB VRAM), and 1 TB of system memory, under Ubuntu 22.04.1 LTS. We implemented MSA-DET in PyTorch 2.3.1 through the Ultralytics YOLO toolbox, with CUDA 12.1 and cuDNN 8.9 handling GPU acceleration. Python 3.10.19 served as the programming environment. Table 3 lists the full hardware and software configuration.

YOLOv11n was selected as the baseline rather than earlier YOLO variants because it incorporates the most recent architectural advances in the nano-scale family—C3k2 modules, C2PSA context-aggregation blocks, and a refined loss—that deliver a better accuracy-to-computation trade-off than YOLOv8n or YOLOv9t at comparable parameter budgets. Our proposed modules are designed as drop-in replacements within YOLOv11n’s backbone and neck, making it the natural architectural foundation.

For HRSID, we follow the official 65/35 train–test split (3643 training images, 1961 test images). A validation set was formed by randomly sampling 10% of the training images (364 images; random seed 42), leaving 3279 images for gradient updates; this validation subset was used exclusively for early stopping and was never included in training. For SSDD, the official random 80/20 partition was applied with the same fixed seed (random seed 42), yielding 928 training images and 232 test images; 10% of the training set (93 images) was similarly reserved for validation. All split indices were fixed prior to any training run so that all compared methods operate on identical subsets.

Input images were resized to 640×640 pixels and fed in batches of 32. Data augmentation during training included mosaic augmentation (combining four images into one), random horizontal and vertical flipping (probability 0.5), and random scale jittering in the range [0.5, 1.5]; no augmentation was applied during validation or testing. At inference, a confidence threshold of 0.25 and a Non-Maximum Suppression (NMS) IoU threshold of 0.7 were used, following the Ultralytics YOLO default configuration.

We used the Adam optimizer [53] with an initial learning rate of 0.002, momentum coefficients $\beta_{1}=0.9$ and $\beta_{2}=0.999$, and weight decay 0.0005. Adam was selected because it combines adaptive per-parameter learning rates with momentum, making it robust to the sparse gradients that arise early in training from scratch on small SAR datasets; the learning rate of 0.002 follows the Ultralytics default for YOLO-family nano models. A linear warmup over the first 10 epochs stabilizes early gradients before the main learning rate schedule takes effect. Training lasted up to 100 epochs, with early stopping triggered if the validation mAP@0.5 did not improve for 10 consecutive epochs. No pre-trained weights were loaded; every model variant started from random initialization to keep comparisons fair and to reflect realistic deployment conditions where ImageNet-pretrained weights may not be available for SAR-specific sensors.

Neither HRSID nor SSDD provides per-image sea-state metadata (significant wave height, Beaufort scale, or equivalent). Both datasets cover a range of maritime conditions acquired over multiple years and orbital passes, so the test sets implicitly include mild-to-moderate sea states. High sea states increase ocean wave returns and surface roughness, intensifying the background clutter that MSA-DET’s speckle-suppression mechanisms (in particular C3k2_SSA’s sparse attention) are designed to address. A controlled sea-state evaluation would require datasets annotated with this information, which we identify as a direction for future work.

### 4.3. Ablation Study

We isolate the contribution of each proposed module—and their joint effect—through ablation experiments on both HRSID and SSDD. Results appear in Table 4 and Table 5. The plain YOLOv11n baseline scores 86.5% mAP@0.5/59.6% mAP@0.5:0.95 on HRSID and 95.6%/62.4% on SSDD. Every variant was trained under identical settings to keep the comparison controlled.

On HRSID (Table 4), adding MSCAttention alone lifts mAP@0.5 to 88.0% (+1.5%) and mAP@0.5:0.95 to 63.0% (+3.4%), confirming that cross-axis multi-scale attention extracts richer features from SAR imagery. C3k2_SSA on its own delivers the largest single-module jump: mAP@0.5 reaches 88.5% (+2.0%) and mAP@0.5:0.95 reaches 63.9% (+4.3%), demonstrating that sparse self-attention in the neck is especially effective at refining features and filtering clutter. Swapping the detection head for ASFF produces a comparable mAP@0.5 of 88.0% and raises mAP@0.5:0.95 to 63.8% (+4.2%), confirming that adaptive spatial fusion tightens bounding-box localization. When all three modules operate together, MSA-DET achieves the highest Precision (91.4%), mAP@0.5 of 88.1%, and mAP@0.5:0.95 of 63.6%. We note that the full model’s mAP@0.5:0.95 is marginally below the C3k2_SSA-only value (63.9%); we attribute this to the interaction between the ASFF weighting and the sparser activations of SSA, which can slightly over-suppress low-confidence small-target predictions under strict IoU thresholds. Critically, the full model achieves the best Precision and the highest mAP@0.5:0.95 simultaneously, making it the most balanced configuration across all four metrics.

The same pattern holds on SSDD (Table 5). The complete MSA-DET configuration achieves 96.6% Precision, 94.1% Recall, 97.7% mAP@0.5, and 69.6% mAP@0.5:0.95, topping every partial combination and confirming that the gains transfer across datasets with different resolution ranges and image sizes.

### 4.4. Comparison with State-of-the-Art Methods

We benchmark MSA-DET against a broad set of competitors on both HRSID and SSDD. The comparison pool spans the transformer-based RT-DETR [54], five successive YOLO generations (YOLOv8n through YOLOv12n) [55,56,57,58,59], and several SAR-specific or small-object detectors: YOLO-MSD [60], FCOS [61], OptiSAR-Net [62], SSMA-YOLO [63], YOLO-LDFI [41], and BL-Net [64]. Every method was trained and tested on the same hardware with the same training protocol.

**Results on HRSID.** Table 6 shows the results. MSA-DET posts the highest mAP@0.5:0.95 at 63.6%, a 4.0% absolute gain over the YOLOv11n baseline. YOLO-MSD does edge ahead on mAP@0.5 (90.2% vs. 88.1%), yet its mAP@0.5:0.95 trails ours by 4.4 percentage points—and it costs 1.4× the parameters (12.3M vs. 8.9M) and 3.5× the FLOPs (33.8 G vs. 9.6 G). RT-DETR lands at 60.7% mAP@0.5:0.95, 2.9% below MSA-DET, while consuming 32M parameters and 108G FLOPs. Against FCOS (84.5%) and OptiSAR-Net (86.9%), MSA-DET holds 3.6% and 1.2% advantages, respectively, in mAP@0.5. OptiSAR-Net is the lightest model in the table at 2.7 M parameters, but that compactness comes with a noticeable drop in detection quality.

**Results on SSDD.** On SSDD (Table 7), MSA-DET leads across the board: 96.6% Precision, 94.1% Recall, 97.7% mAP@0.5, and 69.6% mAP@0.5:0.95. Among SAR-tailored detectors, it surpasses YOLO-LDFI (96.9% mAP@0.5), SSMA-YOLO (89.7%), and BL-Net (91.9%). The gap is widest on the demanding mAP@0.5:0.95 metric, where MSA-DET exceeds the second-best YOLO-LDFI by 3.2 points. YOLO-LDFI does record a higher Recall score of 97.4%, but its looser bounding boxes show up under tighter IoU thresholds—precisely where MSA-DET’s advantage in localization precision becomes evident.

**Inference speed analysis.** We benchmark all models on the same NVIDIA RTX 4090 GPU at 640×640 input resolution with batch size 1, reporting the mean over 1000 inference runs after 200 warm-up iterations. MSA-DET achieves **92 FPS** (10.9 ms latency), which comfortably exceeds the 25 FPS threshold generally accepted for real-time surveillance video and the 30 FPS threshold for broadcast applications. Compared with the YOLOv11n baseline (132 FPS, 7.6 ms), the additional multi-scale attention and ASFF fusion modules introduce a 40 FPS reduction—a reasonable price for 4.0% and 7.2% gains in mAP@0.5:0.95 on HRSID and SSDD, respectively. Among the lightweight YOLO family, MSA-DET is faster than YOLOv9t (55 FPS) and YOLOv12n (79 FPS). For non-YOLO detectors with available implementations, FCOS achieves 69 FPS and RT-DETR-L achieves 12 FPS under the same benchmark conditions; the large latency of RT-DETR-L (85.7 ms) reflects the cost of its transformer decoder. FPS values for models without public implementations (YOLO-MSD, OptiSAR-Net, SSMA-YOLO, and YOLO-LDFI, BL-Net) are marked “–” in the tables.

### 4.5. Visualization of Test Results

Quantitative metrics tell part of the story; visual inspection reveals the spatial character of detection failures and successes. We select dense in-port scenes from the HRSID and SSDD test sets—the hardest case for any SAR ship detector—and compare both bounding-box outputs and feature-level attention heatmaps. Ground-truth bounding boxes in both datasets are the axis-aligned rectangles provided by the respective dataset annotations [7,8]. No re-labeling was performed.

**Figure 6:**
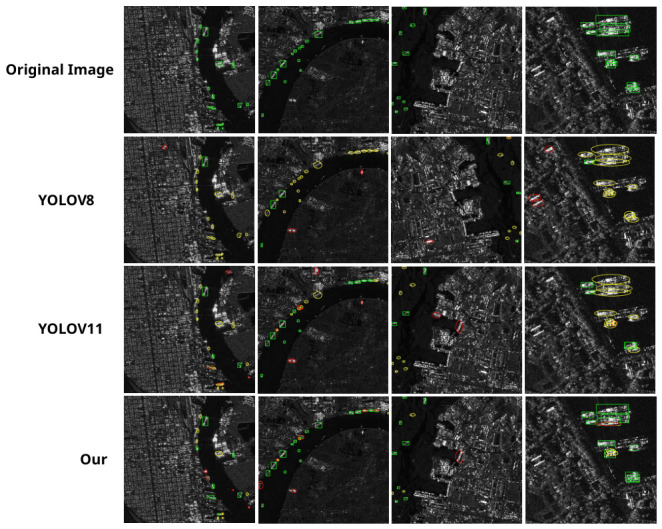
Detectionresults of MSA-DET versus YOLOv11n and YOLOv8n on dense in-port scenes drawn from the HRSID and SSDD test sets. Overlay convention: green boxes indicate true positive detections; red ellipses highlight false alarms (detections with no matching ground-truth ship); yellow ellipses mark missed ships (ground-truth ships with no matching detection). In densely berthed scenarios, multiple annotation boxes and prediction boxes inevitably overlap in the same image area—the colored overlays are drawn together to allow direct visual comparison, not to indicate fused detections.

Figure 6 places MSA-DET side by side with YOLOv11n and YOLOv8n on four crowded harbor scenes. In densely berthed areas, YOLOv11n and YOLOv8n both miss ships and hallucinate detections on dock structures; their predicted boxes frequently merge two adjacent hulls into a single elongated box. MSA-DET cuts down both failure modes: even when the gap between adjacent hulls spans only a few pixels, the ASFF head resolves them as separate detections and the C3k2_SSA-enhanced features keep dock responses near zero.

**Figure 7:**
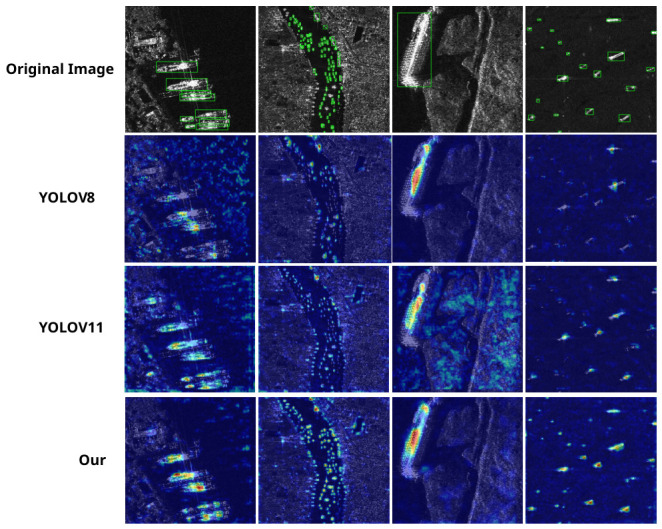
Grad-CAM-stylefeature attention heatmaps for YOLOv11n, YOLOv8n, and MSA-DET on the same in-port test scenes (“jet” colormap: red indicates high activation; blue indicates near-zero activation). MSA-DET concentrates activation tightly on ship bodies and suppresses responses from surrounding water, docks, and buildings, which directly explains its lower false-alarm rate in the detection results above.

Figure 7 compares attention heatmaps rendered with the “jet” colormap (red = high weight; blue = low weight). MSA-DET concentrates activation tightly on each ship body and drops to near-zero over water and shore infrastructure. In the dense berthing case, every vessel receives its own compact hot spot, whereas YOLOv11n and YOLOv8n spread activation diffusely across the scene, frequently highlighting docks and buildings. That diffuse response is precisely what drives false alarms in the baselines; the sharper focus of MSA-DET’s attention maps is consistent with its lower false-alarm rate in the quantitative results.

## 5. Conclusions

SAR ship detection is hampered by speckle noise, cluttered backgrounds, and targets that vary enormously in size. MSA-DET tackles all three issues through a coordinated redesign of the YOLOv11 backbone, neck, and detection head. MSCAttention enriches multi-scale feature extraction via cross-axis information exchange; C3k2_SSA brings sparse self-attention into the neck to suppress noise while preserving global context; and the ASFF head learns per-pixel fusion weights that resolve scale conflicts in the feature pyramid. Experiments on HRSID and SSDD show mAP@0.5:0.95 gains of 4.0% and 7.2% over the YOLOv11n baseline, with parameter and FLOPs budgets that remain moderate. Taken together, these results suggest that end-to-end architectural coordination—rather than isolated module swaps—is a productive direction for SAR-oriented detection.

Several open questions remain. The model has so far been tested on two benchmarks acquired by a limited set of satellites; how well it generalizes to sensors with different frequency bands, incidence angles, or noise profiles is still unclear. The computational footprint, while smaller than that of many competitors, may still be too large for on-board processing on satellites or lightweight edge devices. Going forward, we plan to explore three lines of work: compressing the model through quantization, knowledge distillation, and structured pruning for edge deployment; applying domain adaptation and transfer learning to bridge the gap between different SAR sensors; and optimizing inference throughput via parallel-computing strategies suited to spaceborne hardware. Progress along these directions would bring MSA-DET closer to operational use in applications such as real-time maritime safety monitoring.

## Figures and Tables

**Table 1:** Summaryof representative SAR ship detection methods and their limitations across the three pipeline stages. “✓” indicates the method addresses the corresponding pipeline stage; “–” indicates it does not.

Method	Backbone	Neck	Head	Key Limitation
MSRIHL-CNN [31]	✓	–	–	Fixed hand-crafted features; no multi-scale fusion
MKSFF-CNN [32]	✓	–	–	Patch-based; limited scalability across sensors
YOLOv7-LDS [33]	✓	–	–	Static cross-level fusion; single-scale attention
SED-YOLO [34]	✓	–	–	Limited inter-scale interaction
DETR-axial [35]	✓	–	✓	Heavy transformer backbone; high FLOPs
DSP-YOLO [40]	✓	✓	–	Fixed scale-invariant fusion in head
BiFPN-YOLO [38]	–	✓	–	Spatially uniform fusion weights
YOLO-LDFI [41]	✓	✓	–	No dedicated speckle suppression
**MSA-DET (Ours)**	✓	✓	✓	Moderate parameter increase over baseline

**Table 2:** Detailed parameters of HRSID and SSDD datasets.

Attributes	HRSID	SSDD
Satellites	Sentinel-1B, TerraSAR-X, TanDEM-X	RadarSat-2, TerraSAR-X, Sentinel-1
Polarization Mode	HH, VV, HV, VH	HH, VV, HV, VH
Resolution (m)	0.5–3	1–15
Number of Images	5604	1160
Number of Ships	16,951	2456
Number of Classes	1	1
Image Size (pixels)	800×800	190–526 (Height) 214–668 (Width)

**Table 3:** Experimental environment and training configuration.

Item	Configuration
CPU	Intel Xeon Platinum 8481C
GPU	NVIDIA RTX 4090 (24 GB)
RAM	1 TB
Operating System	Ubuntu 22.04.1 LTS
Programming Language	Python 3.10.19
Deep Learning Framework	PyTorch 2.3.1
Acceleration Library	CUDA 12.1

**Table 4:** Ablation study of proposed modules on the HRSID dataset. “✓” indicates the module is included in the configuration; a blank cell indicates it is not. Bold values denote the best result in each metric column.

Modules				Metrics			
Baseline	MSCAttention	C3k2_SSA	ASFF-Head	P (%)	R (%)	mAP_50_ (%)	mAP_50:95_ (%)
✓				88.6	76.6	86.5	59.6
✓	✓			90.7	78.4	88.0	63.0
✓		✓		90.4	79.1	88.5	**63.9**
✓			✓	90.9	78.4	88.0	63.8
✓	✓	✓		90.7	78.3	87.8	62.5
✓	✓		✓	90.0	78.3	88.0	63.2
✓		✓	✓	89.2	**79.3**	**88.6**	63.5
✓	✓	✓	✓	**91.4**	78.2	88.1	63.6

**Table 5:** Ablation study of proposed modules on the SSDD dataset. “✓” indicates the module is included in the configuration; a blank cell indicates it is not. Bold values denote the best result in each metric column.

Modules				Metrics			
Baseline	MSCAttention	C3k2_SSA	ASFF-Head	P (%)	R (%)	mAP_50_ (%)	mAP_50:95_ (%)
✓				95.0	86.8	95.6	62.4
✓	✓			96.2	91.6	97.0	68.6
✓		✓		95.0	92.3	97.4	68.9
✓			✓	95.9	93.8	97.6	68.3
✓	✓	✓		96.0	91.9	97.4	69.1
✓	✓		✓	95.3	91.8	97.3	68.9
✓		✓	✓	94.6	93.2	**97.8**	69.0
✓	✓	✓	✓	**96.6**	**94.1**	97.7	**69.6**

**Table 6:** Performance comparison of different methods on the HRSID dataset. Params and FLOPs for YOLO-series models are official values at 640×640 input resolution. FPS (frames per second) is measured on a single NVIDIA RTX 4090 GPU at 640×640 with batch size 1, averaged over 1000 runs after 200 warmup iterations; “–” indicates values not available on our hardware. Bold values denote the best result in each metric column.

Model	P (%)	R (%)	mAP_50_ (%)	mAP_50:95_ (%)	Params (M)	FLOPs (G)	FPS
RT-DETR [54]	88.2	**79.8**	86.9	60.7	32.0	108.0	12
YOLOv8n [55]	87.7	77.6	86.7	58.7	3.2	8.1	**176**
YOLOv9t [56]	88.9	76.8	86.6	59.0	2.0	7.7	55
YOLOv10n [57]	84.7	73.9	84.1	57.6	2.3	6.5	110
YOLOv11n [58]	88.6	76.6	86.5	59.6	2.6	6.3	132
YOLOv12n [59]	88.9	75.9	85.9	59.3	2.6	6.5	79
YOLO-MSD [60]	90.2	78.8	**90.2**	59.2	12.3	33.8	–
FCOS [61]	62.9	86.1	84.5	–	32.1	126.0	69
OptiSAR-Net [62]	88.9	76.9	86.9	–	2.7	7.6	–
**Ours**	**91.4**	78.2	88.1	**63.6**	8.9	9.6	92

**Table 7:** Performance comparison of different methods on the SSDD dataset. Params and FLOPs for YOLO-series models are official values at 640×640 input resolution. FPS measurement conditions are the same as for Table 6. “–” indicates values not available on our hardware. Bold values denote the best result in each metric column.

Model	P (%)	R (%)	mAP_50_ (%)	mAP_50:95_ (%)	Params (M)	FLOPs (G)	FPS
YOLOv8n [55]	94.4	90.5	96.0	64.5	3.2	8.1	**176**
YOLOv9t [56]	93.4	89.0	94.9	63.6	2.0	7.7	55
YOLOv10n [57]	88.5	82.1	90.2	58.2	2.3	6.5	110
YOLOv11n [58]	95.0	86.8	95.6	62.4	2.6	6.3	132
YOLOv12n [59]	93.7	86.6	93.7	61.6	2.6	6.5	79
SSMA-YOLO [63]	93.2	89.2	89.7	–	**2.3**	6.8	–
YOLO-LDFI [41]	93.8	**97.4**	96.9	66.4	2.6	6.7	–
BL-Net [64]	91.2	87.0	91.9	–	47.8	417.8	–
FCOS [61]	85.3	86.1	88.7	–	32.1	126.0	69
**Ours**	**96.6**	94.1	**97.7**	**69.6**	8.9	9.6	92

## Data Availability

The HRSID and SSDD datasets analyzed during the current study are publicly available. HRSID is available at https://github.com/chaozhong2010/HRSID (accessed on 15 May 2026) and SSDD is available at https://github.com/TianwenZhang0825/Official-SSDD (accessed on 15 May 2026). The source code and trained model weights for MSA-DET will be made available upon reasonable request to the corresponding author.

## Abbreviations

The following abbreviations are used in this manuscript:

SAR	Synthetic Aperture Radar
YOLO	You Only Look Once
MSA-DET	Multi-Scale Attention Detector
MSCAttention	Multi-Scale Cross-axis Attention
SSA	Sparse Self-Attention
ASFF	Adaptive Spatial Feature Fusion
CFAR	Constant False Alarm Rate
FPN	Feature Pyramid Network
PANet	Path Aggregation Network
mAP	Mean Average Precision
IoU	Intersection over Union
CSP	Cross Stage Partial
SPPF	Spatial Pyramid Pooling - Fast
HRSID	High-Resolution SAR Images Dataset
SSDD	SAR Ship Detection Dataset
FLOPs	Floating Point Operations
FPS	Frames Per Second
